# Molecular Genetic Profile of Myelofibrosis: Implications in the Diagnosis, Prognosis, and Treatment Advancements

**DOI:** 10.3390/cancers16030514

**Published:** 2024-01-25

**Authors:** Tanvi Verma, Nikolaos Papadantonakis, Deniz Peker Barclift, Linsheng Zhang

**Affiliations:** 1 Department of Pathology and Laboratory Medicine, Emory University School of Medicine, Atlanta, GA 30322, USA; 2 Department of Hematology and Medical Oncology, Winship Cancer Institute of Emory University, Atlanta, GA 30322, USA

**Keywords:** myeloproliferative neoplasm, myelofibrosis, primary myelofibrosis, next-generation sequencing, mutations, molecular diagnostics, JAK inhibitor, epigenetic regulation

## Abstract

**Simple Summary:**

Myelofibrosis refers to fibrosis in the bone marrow associated with certain bone marrow cancers. It is a characteristic of primary myelofibrosis and may develop later in other bone marrow cancers with overproduction of blood cells, such as polycythemia vera and essential thrombocythemia. It has been confirmed that mutations in three key genes, Janus kinase 2 (*JAK2*), calreticulin (*CALR*), and myeloproliferative leukemia oncogene (*MPL*), can increase the activity of blood-producing cells, make them grow more actively, and are associated with the development of myelofibrosis. Approximately 80% of myelofibrosis cases carry additional mutations that often involve proteins that control how genes are turned on and off. The presence of mutations provides evidence of a cancerous process. The order in which these mutations occur can influence how the disease manifests. Studies have shown that fibrosis is secondary to the cancerous process and is closely linked to abnormal cell growth driven by mutations. Sophisticated scoring systems have been developed to guide treatment decisions. Specific mutations and genetic changes significantly affect the scores and survival of individual patients. Currently, common treatment involves JAK inhibitors, which can help improve clinical symptoms; however, only a small number of patients show significant alleviation in the biology of the malignant process. New treatments being explored in clinical trials include drugs that target the regulation of genes and substances that modulate the immune system or inflammatory processes. Combining these with JAK inhibitors shows promising results, especially in patients with complex genetic profiles. In the future, by studying more genes, it is expected that researchers will uncover the reasons behind cases where mutations are not found in the three key genes and understand how genetic changes are connected to variable disease presentations, ultimately guiding personalized treatment plans for better outcomes with a chance for cures.

**Abstract:**

Myelofibrosis (MF) is an essential element of primary myelofibrosis, whereas secondary MF may develop in the advanced stages of other myeloid neoplasms, especially polycythemia vera and essential thrombocythemia. Over the last two decades, advances in molecular diagnostic techniques, particularly the integration of next-generation sequencing in clinical laboratories, have revolutionized the diagnosis, classification, and clinical decision making of myelofibrosis. Driver mutations involving *JAK2*, *CALR*, and *MPL* induce hyperactivity in the JAK-STAT signaling pathway, which plays a central role in cell survival and proliferation. Approximately 80% of myelofibrosis cases harbor additional mutations, frequently in the genes responsible for epigenetic regulation and RNA splicing. Detecting these mutations is crucial for diagnosing myeloproliferative neoplasms (MPNs), especially in cases where no mutations are present in the three driver genes (triple-negative MPNs). While fibrosis in the bone marrow results from the disturbance of inflammatory cytokines, it is fundamentally associated with mutation-driven hematopoiesis. The mutation profile and order of acquiring diverse mutations influence the MPN phenotype. Mutation profiling reveals clonal diversity in MF, offering insights into the clonal evolution of neoplastic progression. Prognostic prediction plays a pivotal role in guiding the treatment of myelofibrosis. Mutation profiles and cytogenetic abnormalities have been integrated into advanced prognostic scoring systems and personalized risk stratification for MF. Presently, JAK inhibitors are part of the standard of care for MF, with newer generations developed for enhanced efficacy and reduced adverse effects. However, only a minority of patients have achieved a significant molecular-level response. Clinical trials exploring innovative approaches, such as combining hypomethylation agents that target epigenetic regulators, drugs proven effective in myelodysplastic syndrome, or immune and inflammatory modulators with JAK inhibitors, have demonstrated promising results. These combinations may be more effective in patients with high-risk mutations and complex mutation profiles. Expanding mutation profiling studies with more sensitive and specific molecular methods, as well as sequencing a broader spectrum of genes in clinical patients, may reveal molecular mechanisms in cases currently lacking detectable driver mutations, provide a better understanding of the association between genetic alterations and clinical phenotypes, and offer valuable information to advance personalized treatment protocols to improve long-term survival and eradicate mutant clones with the hope of curing MF.

## 1. Introduction

**Figure 1:**
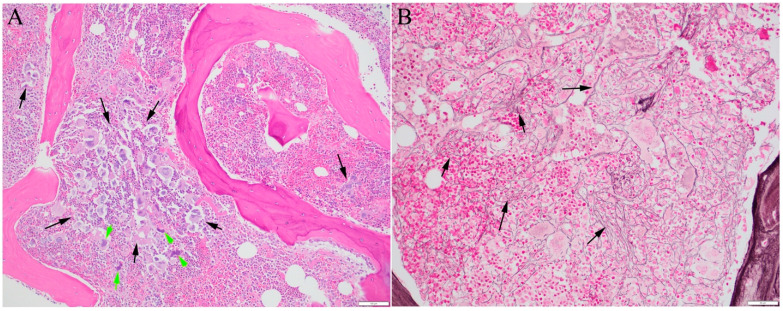
Myelofibrosis (MF, case and images by L.Z.). Bone marrow biopsy images are from a 64-year-old woman diagnosed with essential thrombocythemia (ET) 15 years ago and on intermittent hydroxyurea therapy. (**A**) The hypercellular bone marrow shows frequent atypical megakaryocytes, some displaying hyperchromatic nuclei (green arrows) and forming clusters (black arrows) (H&E stain, 100×, scale: 100 μm). (**B**) Reticulin stain (200×, scale: 50 μm) reveals moderate myelofibrosis (MF grade 2 of 3, representative areas with increased reticulin fiber forming meshwork are indicated by black arrows). Next-generation sequencing of 75 genes associated with myeloid neoplasms revealed *JAK2* V617F at 34.5% and *DNMT3A* R635W at 18.9%. The difference in the variant allele frequency suggests that either the *DNMT3A* mutation is subclonal or the *JAK2* mutation is homozygous. At this stage, the morphologic features and mutation profile of post-ET MF are indistinguishable from those of primary myelofibrosis (PMF).

Myeloproliferative neoplasms (MPNs) are a group of myeloid neoplasms characterized by bone marrow hyperplasia and overproduction of at least one lineage of blood cells. The current subclassification of MPNs is based on changes in blood cell counts, and hematopoietic lineages in the bone marrow that display hyperplasia and dysplasia. Primary myelofibrosis (PMF) is a subtype of *BCR::ABL1*-negative classic MPN, which also includes polycythemia vera (PV) and essential thrombocythemia (ET). The proliferation of abnormal megakaryocytes and varying degrees of fibrosis are defining features of PMF. PMF also typically presents with splenomegaly due to granulocytic proliferation and extramedullary hematopoiesis, and many patients show constitutional symptoms of a hypermetabolic state due to changes in inflammatory cytokines. Recent updates of the 5th edition of the World Health Organization (WHO) Classification of Hematolymphoid Tumors (WHO-HAEM5) [1] and the International Consensus Classification (ICC) [2] have further refined PMF into early, prefibrotic, and overt fibrotic stages. Secondary myelofibrosis (SMF) can present in the later stages of other myeloid neoplasms, particularly other MPNs (post-ET and post-PV MF) and myelodysplastic/myeloproliferative neoplasms (MDS/MPN). It is necessary to differentiate between ET and PV with mild MF and prefibrotic PMF [1]. However, post-PV and post-ET SMF [3,4] can be indistinguishable from PMF when no clear clinical history of PV or ET is documented in patients presenting with myelofibrosis (Figure 1). PMF and SMF are frequently studied together and are clinically managed similarly. Bone marrow fibrosis can also occur in reactive conditions, such as infections, autoimmune disorders, and other malignancies. In the published literature, the term MF is usually reserved for bone marrow fibrosis related to myeloid neoplasms; bone marrow fibrosis is a general term used for other secondary fibrosis [5]. 

MF is a distinctive entity among MPNs, signified by a higher risk of transformation to acute myeloid leukemia (AML). Disease progression of MF can also present as refractory cytopenia, progressive leukocytosis, or refractory progression with an increasing fibrotic burden [6]. With the availability of molecular testing, especially next-generation sequencing (NGS) in clinical laboratories, mutational profiling has transformed the diagnostic and classification paradigms for myeloid neoplasms. Detecting the genetic alterations of MF is not only required for diagnosis as clonal evidence but also provides crucial information to help understand its pathobiology in relation to other myeloid neoplasms. Reflecting the expanding utilization of molecular testing and NGS in clinical laboratories, a bibliometric analysis of publications on MPN from 2001 to 2022 indicated that “gene mutations” has been the top keyword for published studies over the past two decades [7]. However, several aspects of MF remain poorly understood, including the biologic and molecular basis of fibrosis as a distinct feature of PMF, potential biologic distinctions between PMF and post-PV or post-ET MF, and differences between proliferative and dysplastic/cytopenic forms of MF. In this article, we review recent molecular genetic studies related to MF, focusing on mutation profiling-based insights into the pathogenesis and dynamics of clonal evolution of MF, as well as the role of molecular genetics in risk stratification, guiding therapy decisions, and treatment advancements. We hope that this review will provide novel perspectives on the pathobiology of MF and encourage further investigation into personalized treatment based on mutation profiles to improve clinical outcomes.

## 2. Mutation Profile and Clonal Evolution of MF

### 2.1. The Driver Mutations

The discovery of recurrent mutations in Janus kinase 2 (*JAK2*), calreticulin (*CALR*), and myeloproliferative leukemia oncogene (*MPL*) as driver mutations has transformed the diagnostic approach of MPN, as evident in the revisions of WHO classifications [1,8,9]. Clonal evidence, supported by the presence of a driver or other mutations commonly associated with various myeloid neoplasms, is crucial for definitive diagnosis. Both *JAK2* and *MPL* encode proteins that activate the JAK/STAT signaling pathway, which is essential for signal transduction from erythropoietin (EPO), thrombopoietin (TPO), and granulocyte colony-stimulating factor (G-CSF) receptors. The pathobiology and diagnostic relevance of activating mutations in *JAK2*, *CALR*, and *MPL* have been extensively investigated in the clinical setting. *JAK2* V617F mutation is associated with an increased risk of thrombosis, and a high allele burden is associated with disease progression [10]. *MPL* encodes the TPO receptor, and mutations, usually at codon W515, lead to constitutively active signaling independent of ligand binding. The interaction between MPL and altered calreticulin encoded by mutant *CALR* results in MPL hyperactivity [11]. *CALR* and *MPL* mutations are typically exclusive to ET and PMF and very rarely occur in PV [10]; however, *JAK2* V617F mutation remains the most common driver mutation in PMF, reported in 50–60% of cases, followed by *CALR* mutations in 25–35% and *MPL* mutations in 5–10% cases [12,13]. Interestingly, *JAK2* exon 12 mutations [14], which are also activating mutations, have not been documented in ET or PMF. All oncogenic *CALR* mutations are frame-shifting insertions or deletions (indels) that alter the C-terminal end of calreticulin from negatively charged acidic amino acids, aspartic acid (D)- and glutamic acid (E)-rich, to positively charged basic amino acids, arginine (R)- and lysine (K)-rich, removing the endoplasmic reticulin retention signal KDEL. Mutant calreticulin can be secreted and functions as a cytokine, retaining its ability to bind to MPL in the *CALR*-mutated clone [15]. In PMF, type 1 *CALR* mutations (51 bp deletion, L367Tfs*46) are approximately three times more prevalent than type 2 (5 bp insertion, K385Nfs*47) [13], with phenotypic variations observed among *CALR* mutation types [16]. In PMF, type 1 *CALR* mutations correlate with lower leukocytosis, lower bone marrow cellularity, and an increased number of megakaryocytes [13], while type 2 mutations align more closely with the phenotype of cases harboring *JAK2* V617F [17]. 

In the vast majority of MPN cases, driver mutations in *JAK2*, *CALR*, and *MPL* are mutually exclusive. However, there have been occasional reports of cases exhibiting coexistence of *JAK2* V617F, *MPL*, and/or *CALR* mutations [18,19]. Such cases likely involve distinct subclones of neoplastic cells harboring different driver mutations, as demonstrated by a single-cell sequencing study [20], although instances of dual mutations in a single clone have also been documented [21]. Approximately 10% of MPN cases lack detectable canonical mutations in *JAK2*, *CALR*, or *MPL*, categorizing them as triple-negative (TN) MPNs. A small subset of these cases may not truly be TN, as other rare gain-of-function mutations in one of these three genes, particularly *MPL*, have been reported [22,23,24,25,26]. True TN cases often harbor mutations outside of these three genes, confirming clonal hematopoiesis. However, these mutations, which are also prevalent in other myeloid neoplasms, are not considered driver mutations of MPNs. Despite the availability of NGS tests for clinical analysis, the driver mutations of TN cases have not yet been determined, even with comprehensive whole-exome sequencing (WES) studies. One candidate driver, *SH2B3* mutation, has been identified in a subset of TN MPNs [27]. However, *SH2B3* mutations and other driver mutations are not mutually exclusive. The pathogenic drivers of TN MPNs are either heterogeneous non-recurrent mutations, more complicated alterations that evade ready identification by currently available methods, or with mechanisms not yet recognized. Further exploration to understand the regulatory sequences within the non-coding regions of the human genome may shed light on the drivers and molecular pathogenesis of TN MPNs.

### 2.2. Additional Mutations

**Figure 2:**
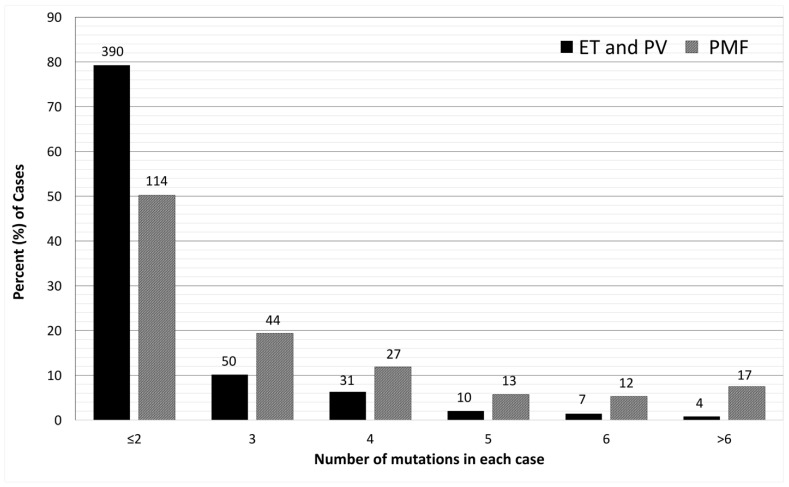
Number of mutations in each sample, essential thrombocythemia and polycythemia vera (ET and PV) versus primary myelofibrosis (PMF). Data source: The AACR GENIE public database [32] (see text for the link to the dataset). ET and PV: 492 samples; PMF: 227 samples. The bar height is displayed as the percentage of samples in each category (Y-axis), and the absolute number of samples in each category is displayed on top of the bar. There is a significantly higher percentage of PMF cases harboring >2 mutations compared with ET and PV cases (49.78% vs. 20.73%, *p* < 0.00001 by Fisher exact test).

With the accumulation of mutation profiling data from clinical studies, it is now clear that over 50% patients with MPNs harbor mutations in addition to driver mutations. Among the classic MPNs, PMF has the highest prevalence of additional mutations. With targeted sequencing of myeloid neoplasm-related genes, additional mutations have been reported in approximately 50% of PV and ET cases, and as high as 80% of PMF cases [28,29,30]. PMF also harbors a higher number of mutations than PV or ET [28,29,31] (Figure 2). Although additional mutations are not considered driver mutations of MPNs, they help establish the clonal nature of TN patients and have been integrated into the major diagnostic criteria of MPNs [1,2]. A query of the American Association for Cancer Research (AACR) Project GENIE public database in cBioportal [32] found 299 samples from 202 cases documented as PMF (https://genie.cbioportal.org/study?id=6562046bb01fff74fbb6c576 (accessed on 25 November 2023)). In these 299 samples, in addition to *JAK2* (44.8%), *CALR* (14.7%), and *MPL* (9.4%) mutations, the prevalence of other mutations is similar to those reported by other studies [29,31,33,34]. Table 1 lists the prevalence of relatively frequent non-driver mutations and the most common mutations or mutation types cataloged in the GENIE database. In addition to the mutations detected in sequencing studies, cytogenetic abnormalities have been reported in 30–57% of PMF cases. However, none of the abnormal karyotypes are specific to PMF [35]. 

The spectrum of additional mutations detected in PMF did not differ from that detected in PV or ET. However, mutations in genes involved in chromosome modification (*ASXL1* and *EZH2*), DNA methylation (*DNMT3A*), and RNA splicing (*SRSF2*, *ZRSR2*, and *U2AF2*) were more frequently observed in PMF [36,37]. Follow-up studies have shown that most somatic mutations in MPN are present at diagnosis, instead of developing during disease progression [38,39]. The mutation profiles were similar in PMF and SMF. *ASXL1* mutation has the highest prevalence, close to 50% in PMF and 30–40% in SMF in some studies [40,41]. Yan et al. studied 258 consecutive PMF patients with 275 samples by sequencing 27 genes, with 17 patients tested on at least two time points, and found that the variant allele frequency (VAF) of *ASXL1* mutations was relatively stable during the disease process [42]. Luque et al. reported that *ZRSR2* and *NFE2* mutations were more common in SMF [41]. 

**Table 1:** Non-driver mutations in primary myelofibrosis.

Gene	Mutation Prevalence (%)	Most Frequent Mutations #	More Frequent in PMF Than Other MPN [34,43]	Clinical Relevance
Epigenetic Regulation (Chromosome Modification and DNA Methylation)				
*ASXL1*	21	Truncation; E635Rfs	Yes	HMR Prevalence increases with age
*DNMT3A*	12	R882H/C	Yes	
*EZH2*	4	Truncation and splice	Yes	HMR
*IDH1/2*	2	*IDH1* R132C/H, *IDH2* R140Q/W	Yes	HMR Prevalence higher in other studies
*TET2*	17	Truncation	No	The order of acquiring mutation affects phenotype
RNA splicing				
*SF3B1*	4	K666N, K700E	No	Associated with ring sideroblasts
*SRSF2*	8	P95	Yes	HMR
*U2AF1*	5	Q157, S34	Yes	HMR
*ZRSR2*	2	Truncation and splice	Yes	More common in SMF [41]
Signal transduction and transcription factors				
*CBL*	6	X366_splice, Y371H	No	Present with other additional mutations [44] Predict poor response to JAK inhibitors [45]
*CUX1*	3	Truncation	Yes	
*NFE2*	2–5 *	E261fs	No, related to erythroid differentiation [25]	Associated with higher risk of transformation to AML, shorter OS. More common in SMF [41]
*NRAS/KRAS*	9	G12	Yes	Relatively specific for MF [25,46]
*RUNX1*	4	Truncation	Yes	Associated with transformation to AML [42]
*SH2B3*	1	Truncation	No	May be considered a driver, or promoting JAK2 activity
*TP53*	2	DNA-binding domain mutations	Yes	Relatively uncommon in MPNs. Associated with higher risk of transformation to AML [39]; however, low VAF in subclone may not increase risk [47]

Abbreviations: HMR: High molecular risk (see the prognostic score section below); MPN: myeloproliferative neoplasm; PMF: primary myelofibrosis; SMF: secondary myelofibrosis, including post-PV and post-ET MF [41]; VAF: variant allele frequency. See footnote for a list of abbreviations for the gene names. Data source: AACR GENIE public database (299 samples from 202 patients; at least 200 samples were studied) [48]. More details can be found at: https://genie.cbioportal.org/study?id=6562046bb01fff74fbb6c576 (need login) (accessed on 25 November 2023). # The mutations are named using a single-letter amino acid code if the most frequent mutations are documented as amino acid changes. * Documented at 0.5% (1/199) in GENIE; prevalence was adjusted based on other studies [49].

MPNs exhibit considerable phenotypic heterogeneity, characterized by variable changes in blood cell counts, presence or absence of dysplastic features and fibrosis, and diverse disease evolution trajectories. However, the biologic ramifications and phenotypic associations with mutation profiles, particularly the impact of additional mutations in individuals with the same driver mutation, remain poorly understood. Studies have indicated that a higher allelic burden of *JAK2* V617F and type 2 *CALR* mutations is correlated with elevated blood cell counts (reviewed by Chifortides et al. [50]). A study by Grinfeld et al. on 2035 MPN patients suggested that genetic mutations and germline polymorphisms contribute, at least partially, to the determination of the phenotype [25]. *JAK2* V617 mutation in the background of *EZH2* knockout mice resulted in a shift in differentiation toward megakaryopoiesis and development of myelofibrosis at the expense of erythropoiesis [51]. *ASXL1* mutation was associated with a unique methylation signature [52]. At the single cell level, subclones with different genetic profiles showed unique transcription signatures [53,54]. However, the biologic effects of these signatures have yet to be characterized.

**Figure 3:**
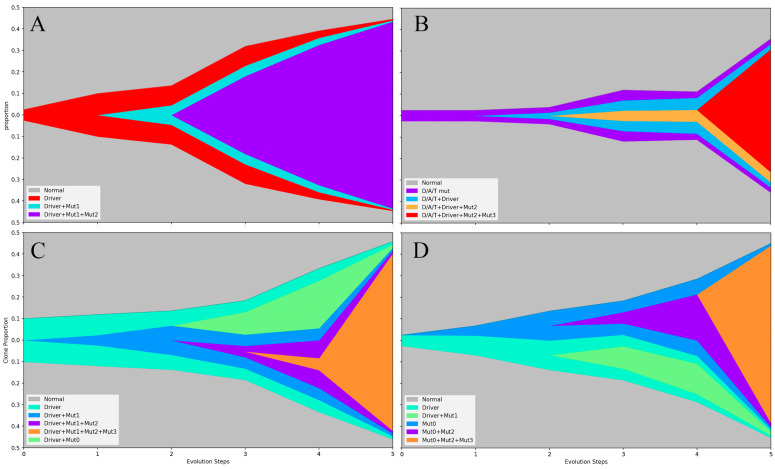
Schematic diagram of the clonal evolution patterns in myelofibrosis (MF). Four patterns of clonal evolution are illustrated based on published clinical and experimental studies. Clones/subclones harboring different mutation(s) are represented in different colors, indicated in the lower left corner of each panel. Most clinical myeloproliferative neoplasm (MPN) cases are likely diagnosed in steps 2–4. More complex and combination patterns exist. The diagram was created using Pyfish 1.0.3 [70] (https://pypi.org/project/pyfish/ (accessed on 6 December 2023)). “Driver” is a driver mutation of myeloproliferative neoplasm (MPN) in one of the three genes: *JAK2*, *CALR*, or *MPL*. (**A**) Linear evolution pattern. Driver mutation emerges from the normal hematopoietic cell population; additional mutations (Mut1, Mut2) acquired stepwise in the same clone with driver mutations. An additional mutation (Mut2) may drive proliferation and expansion of the subclone to become the major neoplastic population. (**B**) Driver mutation acquired in a cell with pre-existing mutation(s). MPN driver mutation acquired in a cell of clonal hematopoiesis of indeterminate significance (CHIP), with mutation(s) represented as D/A/T (*DNMT3A*, *ASXL1*, *TET2*) mut. In addition to the driver mutation, other mutations (Mut2, Mut3) may also be acquired later. (**C**) Branching subclonal evolution pattern. Within a clone with a driver mutation, multiple subclones (driver+Mut1 and driver+Mut0) may coexist, and some may acquire other mutations (Mut2, Mut3) sequentially; after acquiring Mut3, the clone gains a proliferation advantage, driving disease progression or transformation to acute myeloid leukemia (AML). (**D**) Paralleled subclonal evolution. An MPN clone (Driver) and a clone with no MPN driver mutation (Mut0) coexist, multiple subclones may develop from the clone independent of the MPN driver clone, and some (illustrated as Mut0+Mut2+Mut3 clone) may gain a proliferation advantage, becoming the major clone with disease progression or transformation to AML.

A recent study of 216 patients with PMF versus ET/PV found that *KRAS* and *NRAS* mutations were characteristically present in the MF cohort [46], consistent with earlier findings by Grinfeld et al. [25], indicating a strong association between *NRAS* mutations and the MF phenotype. This is also confirmed by a query of the AACR GENIE public database comparing PMF to PV and ET (https://genie.cbioportal.org/study?id=65622d3bb01fff74fbb6c5cc (accessed on 25 November 2023)). *KRAS/NRAS* mutations were found in 17 of 202 (8.4%) PMF patients; however, only 3 of 547 (0.55%) PV and ET patients harbored subclonal *KRAS/NRAS* mutations at low VAFs (<10%). *SRSF2* P95H mutation unexpectedly delayed *JAK2* V617F-associated MF in mouse study [55]. These results highlight the potential influence of additional mutations on the phenotype of MPN and development of MF. Notably, the presence of a clone harboring a *JAK2* mutation independent of those harboring non-driver mutations is not uncommon, and blast transformation of *JAK2*-mutated MPN has been documented in *JAK2* wild-type cells [56] (Figure 3D). While *TP53* mutations are relatively uncommon in MPN and are recognized as a late event [57], a recent study of 349 patients with MF undergoing hematopoietic stem cell transplantation (HSCT) revealed a significantly higher prevalence of *TP53* mutations at 13% [58]. Multiple subclones carrying different *TP53* mutations can coexist within a single patient, further demonstrating a complicated mutational landscape in the late stage of MF. Other genetic alterations associated with transformation to AML are less investigated. A study on samples from 11 patients with MF that progressed to AML, utilizing gene set enrichment analysis (GSEA), reported that samples progressed to AML had increased *E2F* transcription factors. Moreover, in blast phase MPN samples, microRNA *MIR29B1* was upregulated compared to de novo AML [59]. 

### 2.3. The Origin and Evolution of Neoplastic Clones

The sensitive detection of mutations at low VAF made it possible to track the origin and evolution of neoplastic cells carrying specific mutations. A study of *JAK2* mutations across granulocytic, erythroid, and lymphoid lineages revealed heterogeneous stem or progenitor cell origins of MPN in different patients [60]. *JAK2* mutation was detected in myeloid lineage cells in most patients, suggesting an origin from committed myeloid progenitors. However, some patients carried *JAK2* mutation in both myeloid and lymphoid populations, indicating a neoplastic clone potentially originating from multipotent hematolymphoid stem cells [60]. This multipotent stem cell origin appeared to be more common in PMF [61]. Although animal models have demonstrated that driver mutations alone are sufficient to induce MPN [39,62,63,64], *JAK2* V617F has also been identified as an age-related mutation in clonal hematopoiesis of indeterminate potential (CHIP), without resulting in clonal proliferation. Using a sensitive detection method, a Danish study reported *JAK2* V617F in 3.1% and *CALR* mutations in 0.2% of the general population [65]. Cell differentiation studies have confirmed that *JAK2* V617F mutation may not confer a proliferative and/or survival advantage to abnormal clones isolated from PV [60]. Clonal evolution in MPN is a slow process, with a rate of two additional mutations acquired in 133 patient years [38], which reflects the general genomic stability of MPN. Single-colony sequencing of hematopoietic cells from *JAK2*-mutant MPN patients and phylogenetic reconstruction revealed that *JAK2* and *DNMT3A* mutations may be acquired in utero in some patients, persisting in early hematopoietic stem/progenitor cells without terminal differentiation [66]. Significant heterogeneity, with distinct competitive advantages, was observed in patients harboring multiple mutations (Figure 3). When stem/progenitor cells acquire a second mutation and gain proliferation advantage, neoplastic cells grow with lineage-specific expansion [67,68].

A study of patients with MPN carrying both *JAK2* and *TET2* mutations revealed significant differences in expression profiles depending on the order of acquisition of these two mutations [67]. PMF cases were characterized by a higher likelihood of harboring both *TET2* and *JAK2* mutations in the same progenitor cells, suggesting a more complicated mutation profile in the early initiation stages of the malignant clone. Surprisingly, clones carrying two mutations did not exhibit a significant proliferation advantage for expansion. However, the order in which mutations arise affected the composition of hematopoietic populations. When *TET2* mutation preceded *JAK2* mutation, myeloid progenitors predominated, whereas the reverse order resulted in a predominance of megakaryocytic and erythroid progenitors. Moreover, hematopoietic stem and progenitor cells harboring a *TET2* mutation and later acquiring a *JAK2* mutation exhibited a lower proliferative potential. Clinically, patients with a *JAK2*-first mutation tend to be younger and more likely to present with PV and an increased risk of thrombosis. These patients were more sensitive to *JAK* inhibitor therapy. These results are similar to those of another study investigating the order of acquiring driver and *DNMT3A* mutations [69], underscoring the impact of the evolution of mutations on phenotype and clinical presentation. Patients with *TET2* or *DNMT3A* mutations preceding *JAK2* mutation (Figure 3B) were more likely to present with ET. Conversely, acquiring *TET2* or *DNMT3A* mutations in a clone already harboring a driver mutation conferred a proliferation advantage, leading to a higher likelihood of presenting as PV and PMF. These findings suggest a significant effect of mutational dynamics on the pathobiology and clinical presentation of MPN.

Germline genetic background also likely has a significant impact on the emergence and evolution of neoplastic clones in MPN and MF. A large population-based study using the Swedish Family-Cancer Database found that the risk of MPN in individuals with affected first-degree relatives was higher [71]. The *JAK2* 46/1 haplotype and polymorphisms in the telomerase reverse transcriptase gene (*TERT*) have been recognized as genetic factors predisposing to MPN. However, genetic factors or germline gene variants associated with an increased risk of PMF have not yet been well characterized. Pettersson et al. identified a few germline variants in their cohort, involving *CDKN2A*, *NOTCH1*, *ETV6*, and *MPL*, but failed to find obvious clinical relevance [44].

As illustrated in Figure 3, the evolution of mutant clones in MF is heterogeneous. Non-driver mutations may be acquired either before or after the driver mutations. The phenotypes of MPN with or without myelofibrosis are, to some extent, associated with the mutation profile and order of acquiring mutations. The ultimate phenotype is likely determined by the differentiation background of the original neoplastic stem or progenitor cells and their interaction with the microenvironment, which is influenced by the mutation profile. A comprehensive understanding of the pathophysiology of the origin and evolution of different subgroups of MPNs with distinct phenotypes requires further molecular and cellular studies.

### 2.4. Mechanism of Fibrosis

Myelofibrosis, while not exclusive, represents a distinct and essential component of PMF, as a variable degree of fibrosis is required for diagnosis. Myelofibrosis is considered a secondary change that arises indirectly from the neoplastic process. A prevailing hypothesis points to a close relationship between myelofibrosis and neoplastic megakaryocytic hyperplasia with disturbed secretion of inflammatory cytokines. Strong activation of the TPO/MPL/JAK2 signaling pathway is sufficient to induce bone marrow fibrosis in animal models [62,72]. Transforming growth factor-beta (TGFβ) [73] and platelet-derived growth factor (PDGF) [74], which are synthesized by megakaryocytes, have been recognized as major mediators of myelofibrosis. TGFβ stimulates the synthesis of extracellular matrix components, such as collagens, fibronectin, tenascin, and proteoglycans. Simultaneously, it inhibits matrix degradation by reducing proteases, effectively promoting fibrosis [73]. The significance of TGFβ-1 in the fibrogenic process of PMF was demonstrated in a TGFβ-1^−/−^ animal model, where overexpression of thrombopoietin (TPO) failed to induce myelofibrosis, in contrast to the severe bone marrow fibrosis developed in control wild-type mice. Moreover, TGFβ-1 plays an important role in regulating the inhibitory factor osteoprotegerin (OPG) and contributes significantly to the osteosclerosis observed in late-stage PMF [75,76]. Upregulation of TGFβ in mutant megakaryocytic progenitors, correlating with the degree of myelofibrosis, has been confirmed by a study at the single cell level [53]. However, clinical studies examining bone marrow samples from patients have failed to establish a direct correlation between increased TGFβ-1 expression and the degree of myelofibrosis in MPN [75].

Both animal models and patient samples have highlighted the important role of additional inflammatory factors such as IL1, IL4, and IL13, in the promotion of myelofibrosis [77,78,79]. Activation of the NF-κB pathway via mutant JAK2 has been proposed to be a key contributor to inflammatory processes in MPNs. This is further supported by the clinical observation that treatment of MF with the JAK inhibitor ruxolitinib reduces cytokine levels and MF grades, at least in a subset of patients. Lysyl oxidase (LOX) plays an important role in crosslinking collagen and elastin in an array of different tissues [80]. In the context of myelofibrosis, studies in mice have demonstrated its expression in low ploidy megakaryocytes. Inhibition of LOX ameliorated fibrotic burden in a mouse model using the deaminase inhibitor β-aminopropionitrile (BAPN) [81]. However, BAPN’s array of toxicities limits its application in clinical practice. In addition to LOX, other LOX-like (LOXL) proteins have been implicated in the pathogenesis of myelofibrosis [82]. Importantly, several inhibitors with different specificities for LOX and LOXL proteins are being tested in clinical trials [83].

Although the role of megakaryocytes in the development of fibrosis has been extensively studied, other hematopoietic cells may also play an important role. Fibrocytes originate from neoplastic monocytes and exhibit fibroblast-like features. Fibrocytes have many attributes, including the secretion of cytokines and modulation of fibrosis. Bone marrow samples derived from patients with PMF were enriched with fibrocytes compared to samples not affected by MPN. The pentraxin protein serum amyloid P (SAP) can effectively inhibit fibrocyte formation. Experiments with xenotransplantation of bone marrow cells from patients harboring *JAK2* V617F and chromosome abnormality del(20q) demonstrated that recombinant SAP administration inhibited the development of fibrotic burden and proliferation of fibrocytes [84]. Notably, in vitro differentiation of fibrocytes was not affected by ruxolitinib [84]. In another series of experiments using mouse models overexpressing *JAK2* V617F, the depletion of monocytes resulted in a marked decrease in the number of fibrocytes, reversal of reticulin fibrosis, and amelioration of collagen fibrosis in the bone marrow [85]. Moreover, splenomegaly was ameliorated as well as the splenic fibrosis. However, depletion of monocytes and improvement of fibrotic burden did not translate to the resolution of megakaryocytic lineage expansion. Interestingly, in these mouse models, leukocytosis and increased platelet count persisted [85]. The modulation of monocyte differentiation and cellular fate in myelofibrosis remains largely unknown. Studies have implicated changes in viscoelasticity and extracellular matrix deposition as potential contributors [86].

The mechanisms underlying phenotypic variations among MPNs with a similar mutation profile require further study, as reviewed by Ghosh et al. [87]. The VAF of the driver mutations may play a significant role. Even in patients at the prefibrotic stage of PMF, the *JAK2* V617F mutation burden is higher than that in ET [88,89]. An identical mutation carried by cells at different stages of differentiation may exhibit distinct phenotypes that are influenced by chromosome accessibility and the epigenetic landscape of the neoplastic cells [90,91]. A single-cell transcriptome and proteomics study [92] found that hematopoietic stem/progenitor cells from PMF harboring *JAK2* V617F showed megakaryocyte-biased hematopoiesis with a more frequent expression of G6B, a surface marker exclusively expressed on mature megakaryocytes in normal hematopoiesis. The role of *CALR* mutations in the development of myelofibrosis is complex as the same mutation can be seen in other MPNs. While mutated calreticulin interacts with MPL, resulting in hyperactivity of the MPL signaling pathway, secreted mutant calreticulin can have an immunomodulatory role and suppress dendritic cell function [93]. A recent study [94] reported that human hematopoietic stem cells (HSC) engineered through CRISPR/Cas9 technology and adenovirus-associated vector knock-in approach to express mutant calreticulin uncovered skewing towards the megakaryocytic lineage and compensatory increase of other chaperones. The xenotransplant mouse model of these engineered HSC expressing mutant calreticulin developed splenomegaly and myelofibrosis. Such models may facilitate the discovery of the mechanisms that lead to mutant calreticulin-mediated myelofibrosis. However, the effects of additional mutations on the emergence of myelofibrosis have not been well studied. Some experts have proposed that PMF represents a presentation in the advanced phase of a previously undiagnosed MPN [12]. In patients diagnosed with ET or PV, the presence of reticulin fibrosis in the bone marrow, although associated with an increased risk of transformation to SMF, was not associated with shorter survival [95,96]. Therefore, distinguishing PV and ET with mild MF from prefibrotic stage PMF has a significant clinical and prognostic value.

### 2.5. Laboratory Test Considerations

Despite the wide availability of NGS-based targeted gene sequencing, point mutation tests for *JAK2* V617F and single gene tests for *CALR* exon 9 indels are still routinely performed in many clinical laboratories owing to their cost-effectiveness and high diagnostic yield, particularly for the initial diagnosis of MPN. Polymerase chain reaction (PCR)-based *JAK2* point mutation tests offer the advantage of high analytical sensitivity that can detect mutations at a VAF as low as 0.1%. The digital droplet PCR method can achieve a sensitivity lower than 0.01%. Other new approaches can also be used to detect *JAK2* V617F mutation at very low levels. One study reported the detection of the *JAK2* V617F mutation at a level of 0.01% using a CRISPR/Cas12a based approach [97]. These sensitive methods are particularly suitable for post-treatment follow-ups. To cover a diverse spectrum of over 50 *CALR* indels, single-gene tests for *CALR* mutations typically rely on fragment length analysis (FLA), melting curve analysis, or conformation sensitive gel electrophoresis (CSGE) of PCR amplicons. Pathologists and oncologists need to thoroughly understand the potential pitfalls associated with these tests, especially when interpreting negative results.

Although *JAK2* c.1849G>T (V617F) is the most common mutation reported in MPN, other rare mutations affecting codon V617 have been documented. In the Catalogue Of Somatic Mutations In Cancer (COSMIC) database (https://cancer.sanger.ac.uk/cosmic, accessed on 6 December 2023), *JAK2* c.1849G>T; p.V617F (COSV67569051) is documented in 42886 of 54279 (79%) unique samples with *JAK2* mutations, *JAK2* c.1849G>A; p.V617I (COSV67571909) is documented in 89 (0.16%) samples, and the double/multiple nucleotide mutations c.1848_1849delinsCT; p.V617F (COSV67586666), c.1849_1852delinsTTCC; p.V617_C618delinsFR (COSV67578410), and c.1849_1852delinsTTTC; p.V617_C618delinsFR (COSV67606858) are documented in rare cases. These mutations likely result in similar activation changes in JAK2 but present a challenge for laboratory detection. Allele-specific PCR for *JAK2* point mutations may yield unusually low mutant percentages or negative results for these atypical variants. Although the highly sensitive *JAK2* point mutation test is excellent for post-treatment follow-up, the development of an equally sensitive test for *CALR* mutations is a formidable challenge because of the diversity of *CALR* mutations. Allele-specific PCR designed for types 1 and 2 mutations can detect >80% of *CALR* mutations. To cover all exon 9 indels of *CALR*, the analytic sensitivity of a single gene test based on FLA or melting curve analysis is approximately 5% VAF. The analytic sensitivity of CSGE has not been well defined [98,99], but it may not be significantly better than melting curve analysis. Therefore, *CALR* single gene tests are primarily suitable for initial diagnosis. In addition, benign germline *CALR* in-frame variants within exon 9 have been well documented [100]. It may not be easy to distinguish benign in-frame deletions from oncogenic indels because of inaccurate size calibration using FLA. In challenging cases, germline DNA analysis may be required [101].

Another observation from *JAK2* mutation tests is the possibility of unusually low *JAK2* allele frequencies in the peripheral blood of certain MPN patients. This phenomenon may have resulted from mutant clones skewed toward erythroid and megakaryocytic lineages. Terminally differentiated red blood cells or platelets in peripheral blood are anucleate, and the DNA used for mutation testing is extracted from nucleated granulocytes or lymphocytes that harbor no mutation [102]. Therefore, in cases where *JAK2* mutation test results appear inconsistent with clinical features or when there is a suspicion of benign *CALR* indels, it is advisable to consider an NGS-based test using a bone marrow sample. Given the existence of unusual *JAK2* mutations and the high prevalence of additional mutations beyond driver mutations in MF, routine incorporation of NGS-based mutation profiling at the time of diagnosis is imperative. Bioinformatics pipelines for NGS data analysis are known to exhibit variable accuracies in detecting and naming large indels [103]. Modifying the bioinformatics pipeline for large indel detection is essential when devising NGS-based mutation profiling tests for myeloid neoplasms. With the decreasing costs of massive parallel sequencing and improvement in the quality of long-read sequencing technology, it is plausible that whole-exome sequencing (WES) and whole-genome sequencing (WGS), complemented by whole-transcriptome sequencing (WTS), will transition into routine clinical tests in the near future. These comprehensive tests hold promise in unveiling novel oncogenic alterations, thereby providing a deeper understanding of the pathobiology underlying myelofibrosis [104,105].

## 3. Prognostic and Therapeutic Implications of Mutation Profiles

### 3.1. Implications in the Prognosis

Prognostic stratification for MF began with the development of the International Prognostic Scoring System (IPSS) in 2009, primarily based on clinical and laboratory features [106]. As molecular genetic testing became widely available in clinical laboratories, clinical studies quickly demonstrated that certain abnormal karyotypes and mutations independently correlated with clinical outcomes. A sophisticated scoring system, the Mutation-Enhanced International Prognostic Scoring System plus version 2.0 (MIPSS70 + v2.0, Table 2) for PMF was established in 2018 [107]. This advanced system integrated both cytogenetic and mutation profiles and built upon the Mutation-Enhanced IPSS (MIPSS70) [108]. Notably, the absence of *CALR* type 1 mutation is considered a risk factor, and mutations in *ASXL1*, *EZH2*, *SRSF2*, and *IDH1/2* are categorized as high-molecular risk (HMR) mutations. In a separate genetically inspired prognostic scoring system (GIPSS), cytogenetic abnormalities were included as important prognostic factors, and *U2AF1* Q157, together with non-*CALR* type 1, *ASXL1*, and *SRSF2* mutations, were added as HMR mutations [109]. Risk stratification by cytogenetic abnormalities was three-tiered: a very high risk (VHR) group included single/multiple abnormalities of inv(3)/3q21, −7, 11q−/11q23, 12p−/12p11.2, i(17q), +21, or other autosomal trisomies, excluding +8/+9; a favorable group included normal karyotype or sole abnormalities of translocation or duplication involving chromosome 1, +9, 13q−, 20q−, or sex chromosome abnormality including -Y; and all other abnormalities were grouped as high-risk (HR) [109]. According to WHO-HAEM5, a myeloid neoplasm with inv(3)/3q21 is classified as AML with defining genetic abnormalities, regardless of the blast percentage [1]. The evolving landscape of prognostic systems for myelofibrosis underscores the increasing recognition of complex clinical, cytogenetic, and molecular factors for predicting disease outcomes. The prognostic value of HMR mutations in MIPSS70+ V2.0 has been confirmed by recent clinical study results [110,111]. HMR mutations, especially *ASXL1* G646Wfs*12, *RUNX1* L56S, *ZRSR2* R169*, and *U2AF1* Q157P, were also associated with the progression of PV and ET to SMF. A separate prognostic scoring system, Myelofibrosis Secondary to PV and ET-Prognostic Model (MYSEC-PM) [112], was established for SMF, in which only the *CALR* unmutated genotype was considered a high-risk factor [112].

Although not represented in the HMR mutations, TN MPN (including MF) had worse prognoses in some study cohorts [4,17,115,116]. Additionally, various studies have revealed prognostic associations with other mutations. *ASXL1* is the second most frequently mutated gene in MF after *JAK2* V617F and has demonstrated significant associations with progression from prefibrotic to overt PMF, transformation to accelerated and blast phases, and an overall worse prognosis [42,113]. Notably, mutations in *ASXL1* are frequently observed in CHIP, which increases with age. Contrary to earlier findings, Petterson et al. reported that when age was taken into consideration, the presence of *ASXL1* mutations no longer correlated significantly with worse overall survival (OS) in MF [44]. Another study by Luque et al. indicated that, when present as a solitary mutation without additional high-risk mutations, *ASXL1* mutations were not indicative of a poor prognostic outcome in MF [41]. *TP53* mutations, a well-recognized indicator of poor prognosis in MDS and AML, were also not included in HMR mutations. Although usually emerging as late-stage mutations, clones with *TP53* mutations may become the dominant neoplastic population, leading to transformation to acute leukemia [25,57]. The different conclusions regarding the prognostic significance of *TP53* mutations in MPN are likely related to the stage at which the mutation profiling was performed. At diagnosis, the prevalence of *TP53* mutations may not be high enough to be statistically significant. *RUNX1* mutation has been linked to inferior survival or transformation to acute leukemia in a few studies [29,39,42]. In a study of 363 patients with PMF, mutations in *CBL*, *NRAS*, *KRAS*, *RUNX1*, and *TP53* did not show significant prognostic value [110]. In another analysis of recurrent mutations in a cohort of 248 MPN patients [44], mutations in only five genes, *ASXL1*, *SRSF2*, *U2AF1, CBL*, and *SF3B1*, were associated with inferior OS, regardless of the type of MPN. *CBL* mutations probably did not have an independent prognostic value, because all cases with *CBL* mutations also harbored a mutation in one of the other four genes. When analyzed separately and adjusted for age and type of diagnosis, only *SRSF2* and *U2AF1* mutations remained significantly correlated with OS. Mutations involving these two genes were also associated with the progression of PV and ET to SMF and the development of other myeloid neoplasms (CMML and MDS), with only *SRSF2* being significantly associated with AML transformation.

Grinfeld et al. [25] sequenced 69 myeloid neoplasm-related genes in 2035 patients with MPN (148 patients had WES results), including 309 patients with MF, and found that specific driver mutations, genetic background of germline polymorphisms, and patient demographic variables independently predicted MPN classification. Eight genomic subgroups were evident from the study, with distinct clinical phenotypes, risk of leukemic transformation, and event-free survival. Eventually, a Personalized Risk Calculator for MPN [25], independent of MIPSS70+, was established based on 63 clinical and genomic variables to predict clinical outcomes in patients with MPN and MF (Table 2). This model was validated using an external cohort of 515 patients with MPN, including 190 patients with MF. Although they played a substantial role in PV or ET progression to MF and MPN transformation to AML, the genomic features were not significantly associated with survival in chronic-phase MPN without MF. Unsurprisingly, there were no significant differences in the survival of patients with PMF or SMF. This study further supports the concept that genetic abnormalities are pivotal in the pathogenesis of MPN and MF.

It is undeniable that the current molecular genetic profiling results fall short of capturing all prognostically significant factors in MF. Beyond the IPSS, additional parameters such as spleen size and levels of lactate dehydrogenase (LDH) and ferritin [117] have been linked to the risk of disease progression and OS. Conversely, the Myelofibrosis Transplant Scoring System (MTSS, see Table 2), established to aid in selecting patients for HSCT, considers only the non-*CALR/MPL* driver mutation genotype and *ASXL1* mutation as risk factors. However, *TP53* mutations have been recognized as a negative prognostic factor in patients who have undergone HSCT in other studies [118]. A recent investigation involving transplant patients confirmed that multi-hit *TP53* mutations were associated with a higher risk of relapse and reduced overall survival [58]. High-sensitivity molecular methods to detect a patient’s known mutations may serve as minimal residual disease (MRD) monitoring tests to identify patients with a high risk of relapse [119]. Based on the findings of Grinfeld et al. [25], the subclassification of PV and ET demonstrated limited prognostic value in distinguishing between MF and MPN without MF. Consequently, it is plausible that in the future, molecular genetic profiling may assume a more prominent role in the prediction of prognosis and treatment response, superseding reliance on clinical phenotypes.

### 3.2. Treatment Implications

Currently, MF treatment aims to alleviate symptoms, reduce the burden of splenomegaly, slow disease progression, and prevent the transformation to acute leukemia. The treatment strategy depends on the clinical presentation, risk stratification, prediction of prognosis, and transplant-specific risk (MTSS) to select patients eligible for HSCT. HSCT is the only curative treatment available for eligible patients. Given that most DIPSS low- and intermediate-1 patients do not require treatment [5], accurate risk stratification with cytogenetic and mutation profiling tests is essential for treatment decisions. The approval of JAK inhibitors and other novel agents has significantly changed the treatment landscape of MF. Ruxolitinib is currently the standard of care for high-risk MF, including both PMF and SMF. Ruxolitinib has been proven not only to alleviate the symptoms, and effectively reduce spleen size, but also to lower the level of blood cytokines, and possibly increase the overall survival although the conclusion remains controversial [120,121]. Three additional small-molecule kinase inhibitors have been approved by the United States Food and Drug Administration (US FDA) for the treatment of MF [122,123] (Table 3). JAK inhibitors are not mutation-specific; therefore, *JAK2* mutations are not a required selection criterion for treatment. However, a higher *JAK2* mutant VAF has been associated with a better splenic response to ruxolitinib [124].

Reduction of *JAK2* mutant allele burden has been proposed as a surrogate for treatment effectiveness. Multiple clinical trials have suggested that a >20% reduction in VAF is associated with a higher rate of spleen response, symptomatic improvement, decreased fibrosis level, and longer overall survival [125]. Overall, ruxolitinib as a single agent in frontline therapy has not demonstrated a consistent biological effect in reducing the mutation burden [126]; the improvement in survival is significant in MF patients with a high mutation burden [124], but overall still controversial [125,127]. Discontinuation due to failed response, disease progression, or intolerance is up to 60% of patients within 3 years [121,128,129]. During MF disease progression, most patients develop cytopenia, usually anemia or thrombocytopenia. Although both ruxolitinib and fedratinib may effectively reduce the *JAK2* V617F allele burden [130], they also contribute to the suppression of erythropoiesis and thrombopoiesis. Therefore, their indications may be limited owing to preexisting cytopenias. Approximately 25% of patients who discontinued ruxolitinib therapy were due to ruxolitinib-related cytopenias or infections [128]. New small-molecule kinase inhibitors with inhibitory effects on ACVR1, pacritinib and momelotinib, were developed to address this issue. ACVR1 mediates hepcidin production in the liver, resulting in decreased serum iron availability. ACVR1 inhibition is helpful in improving erythropoiesis during MF treatment, and is therefore suitable for anemic patients [131,132]. Notably, pacritinib responses for splenic volume reduction were superior to the best available therapy when stratified by the absence of *JAK2* V617F or low *JAK2* V617F allelic burden (up to 50% allele frequency) [133]. Symptom responses followed a similar pattern (for allelic frequency of *JAK2* V617F between >0% and 50%) [133]. Momelotinib was able to reduce *JAK2* V617F allelic burden from baseline in patients participating in phase 1/2 utilizing a twice daily dosing [134]. More clinical data are required to determine the effect of these newer JAK inhibitors on *JAK2* mutation burden and long-term survival in patients with cytopenic MF.

Several studies have demonstrated that additional non-driver mutations may predict clinical response to JAK inhibitor treatment. The epigenetic regulators *ASXL1* and *EZH2* have been identified as predictors of poor response to ruxolitinib [135,136]. Mutations in splicing factors *SRSF2* and *U2AF1* [107] and genes of RAS/MAPK pathway (*NRAS, KRAS, CBL*) have also been associated with resistance, intolerance, or shorter OS in some studies [45,46,137,138]. Multiple (>3) mutations have also been associated with poor response and shorter OS [135]. It is not surprising that clonal evolution occurred in the patients receiving JAK inhibitor therapy [139]. Under selection pressure, emerging clones are more likely to be drug-resistant. *ASXL1*, *TET2* [139], and RAS pathway mutations [140] have been documented as the most frequent emerging mutations during ruxolitinib treatment. Clonal evolution during the treatment process indicates the need to monitor the mutation profile in the follow-up of patients treated with JAK inhibitors. A study from the Mayo Clinic, including patients treated with momelotinib, identified the absence of *CALR* type 1 and the presence of *ASXL1* and *SRSF2* mutations as factors adversely affecting survival (including leukemia-free survival) [141].

JAK inhibitors have not been able to eradicate neoplastic clones, as seen in targeted therapy with tyrosine kinase inhibitor (TKI) treatment for *BCR::ABL1*-positive chronic myeloid leukemia (CML). Given the frequent mutations of genes involved in epigenetic regulation, including chromosome remodeling, DNA methylation, and RNA splicing in MF, clinical trials with a variety of agents targeting epigenetic regulation in combination with JAK inhibitors are ongoing and appear to show promising results. An in vitro study showed that the pan-HDAC inhibitor panobinostat, together with a JAK inhibitor, effectively depleted JAK/STAT signaling and synergistically induced apoptosis of cells harboring the *JAK2* V617F mutation [142]. However, clinical trials of this combination have found that the overall response rate was not significant enough to support further development, and there were also relatively more cytopenic side effects [143]. Newer HDAC inhibitors are required before this treatment can be revived in clinical settings. Lysine-specific demethylase 1 (LSD1) is a H3K4 demethylase that functions as a transcriptional regulator. In an animal model study, an LSD1 inhibitor effectively synergized with ruxolitinib, lowered *JAK2* V617F mutant allele burden, and improved survival [144]. It has also been found to selectively inhibit *ASXL1*-mutant clone and to be more effective in *JAK2* than in *CALR*- or *MPL*-mutated cells [145,146,147]. Given the role of LSD1 as an epigenetic regulator and its effect on *ASXL1* clones, it is reasonable to further study whether the treatment effect is associated with other epigenetic regulator mutations observed in a subset of myelofibrosis in clinical settings.

Bromodomain and extra-terminal proteins (BET) also function as epigenetic regulators by interacting with acetylated lysine on histones to regulate gene expression. BET inhibitors have shown antiproliferative, anti-inflammatory, and antitumor effects in in vitro and animal studies (reviewed by Palumbo and Duminuco [148]). Pelabresib, a pan-BET inhibitor, in combination with ruxolitinib, has been shown to lower inflammatory cytokines, act on megakaryocyte differentiation and proliferation, and decrease fibrosis in clinical trials [149,150,151]. A recent study revealed that cells harboring *CALR* mutations might be more sensitive to BET and HDAC inhibitors [152]. Protein arginine methyltransferase 5 (PRMT5), phosphorylated by *JAK2* V617F, methylates both histone and non-histone proteins. A study of patient samples revealed higher PRMT5 expression in MPN with *JAK2* V617F mutation. An in vitro and mouse model study showed that a PRMT5 inhibitor effectively suppressed the proliferation of cells harboring *JAK2* V617F, and when combined with ruxolitinib, the PRMT5 inhibitor C220 showed a better effect on lowering the mutation burden, reducing blood cell counts and spleen size in both *JAK2* V617F and *MPL* W515L animal models [153]. Clinical trials of the PRMT5 inhibitor PRT543 on MF are ongoing and appear to show promising responses. PRT543 is currently being evaluated as a monotherapy for MF and MDS patients with at least one spliceosome gene mutation. One patient harboring an *SF3B1* mutation experienced substantial improvement in anemia [154]. Another new agent that has demonstrated promising results in clinical trials for MF (DIPSS intermediate-2 or high-risk) refractory to JAK inhibitor treatment is the telomerase inhibitor imetelstat [155]. Treatment response was affected by additional mutations in a pilot study [156]. Further studies are required to determine the efficacy of imetelstat for clearing neoplastic clones [157].

The BCL2 inhibitor venetoclax has been effective in a subgroup of patients with MDS and AML. In MPN, *BCL-xL* and *BCL2* are overexpressed as targets of upregulated *STAT5*. The BCL2 and BCL-xL inhibitor ABT-737 worked synergistically with a JAK inhibitor and effectively overcame acquired ruxolitinib resistance in an animal experiment [158]. Clinical trials of combination therapy with BCL2, BCL-xL, and BCL-W inhibitor navitoclax showed promising results in most patients, with significant symptomatic alleviation, decrease of spleen size, improvement in anemia, lowering of blood cytokine levels, reduction of fibrosis burden (at least 1-grade reduction, with some achieving complete resolution), and at least half of the cases achieving >20% *JAK2* or *CALR* mutant allele reduction (reviewed by Pemmaraju et al. [125]). The combination achieved a good response in approximately 30% of the patients with relapsed or refractory MF. More importantly, the clinical response and reduction in mutation burden by the combination therapy of either BET or BCL2/BCL-xL inhibitor with ruxolitinib were not affected by additional HMR mutations, indicating that combining pelabresib or navitoclax with ruxolitinib is as effective in patients with HMR mutations as in those with only a simple mutation profile. These exciting clinical trial results from novel agents or combinations suggest a promising new approach for the treatment of MF patients harboring HMR mutations. Results of navitoclax and ruxolitinib vs. ruxolitinib with placebo phase 3 clinical trial for JAK inhibitor treatment-naïve MF patients (Transfom-1) were recently presented at the ASH conference 2023. The combination achieved a primary endpoint spleen volume reduction of ≥35% at week 24 in 63% of patients compared to 31% in the ruxolitinib arm. However, the mean total symptom score change from baseline was not statistically significant between the two arms [159]. Similar results were reported in the phase 3 placebo-controlled MANIFEST-2 trial, which randomized JAK inhibitor treatment-naïve MF patients to pelabresib and ruxolitinib vs. ruxolitinib and placebo [160]. The endpoint spleen volume reduction of ≥35% at week 24 was achieved in 66% of patients in the pelabresib combination group versus 35% in the ruxolitinib and placebo group. The total symptom score was reduced in both arms but statistical significance was borderline (*p* = 0.054). Patients with MF harboring *IDH2* mutations showed a significant response to a combination of IDH2 inhibitor enasidenib and ruxolitinib [161]. This clinical trial required the *IDH2* mutation as a prerequisite. It would be interesting to investigate whether a better response to novel agents targeting epigenetic regulators is related to mutations in epigenetic regulatory genes.

Dysregulation of inflammatory cytokines is a significant pathobiological process in MF. JAK inhibitors are considered anti-inflammatory drugs that have been successfully used in the treatment of rheumatoid arthritis, inflammatory bowel diseases, and COVID-19 [162]. However, treatments that specifically target inflammatory processes have not achieved significant progress in MF [163,164,165]. On the other hand, interferon alpha (IFN-α) is currently used for ET and PV and has been found to effectively suppress neoplastic clone [166] in a driver mutation-dependent manner [167,168], presumably affecting the proliferation and differentiation dynamics of hematopoietic stem/progenitor cells harboring mutations [169,170], thus depleting the neoplastic clone at the stem cell level. IFN-α has been used in the context of myelofibrosis in limited patient series. In a study focusing on early myelofibrosis (DIPSS low- or intermediate-1), baseline driver mutations did not affect the treatment response, but the presence of *ASXL1* or *SRSF2* had a deleterious impact [171]. Although limited by sample size (*n* = 4), patients with *SRSF2* or *ASXL1* mutations did not respond well to IFN-α. However, 37% of patients attained partial or complete remission [171]. Combining the hypomethylating agent (HMA) 5-azacytidine with pegylated IFN-α (pegifna) significantly suppressed the neoplastic clone in a mouse model with *JAK2* V617F and loss of *Dnmt3a* [172]. A clinical trial of the ruxolitinib and pegifna combination in patients with MF also demonstrated excellent efficacy [173]. The primary efficacy endpoint of spleen length reduction of at least 50% within 24 weeks was reached in 70% of patients, including complete resolution of palpable splenomegaly in 38% of patients. *JAK2* V617F allele burden decreased by a median of 31% after 12 months of treatment. The combination of pegifna and ruxolitinib was confirmed to target progenitors carrying the *JAK2* V617F mutation by genotyping progenitor-derived colonies [173].

Immunomodulatory drugs such as pomalidomide can effectively modulate inflammatory cytokines and have been shown to improve anemia and thrombocytopenia and reduce spleen size in patients with MF. A better treatment response is associated with -5q and *JAK2* V617F mutation [5]. A retrospective study of 176 patients who received lenalidomide or thalidomide treatment revealed a relatively high prevalence of mutations in the spliceosome genes. Except for *SRSF2* mutations (only four patients), the other mutations did not appear to affect the clinical benefit of treatment with immunomodulatory drugs [174]. These findings suggest that a combination of JAK inhibitors and HDAC, HMA, IFN-α, or immunomodulatory drugs may effectively suppress neoplastic clones in patients with HMR mutations and exhibit MDS-like or cytopenic phenotypes. Drugs and combination therapies with established efficacies and promising clinical responses are summarized in Table 3.

**Table 3:** Established and promising novel therapies for myelofibrosis.

Drug	Target/Mechanism	Indications, Clinical Study Findings
JAK inhibitors, approved by US FDA		
Ruxolitinib	JAK1/2	Approved for intermediate and high risk PMF or SMF [121]
Fedratinib	JAK2 and FLT3	Similar to ruxolitinib [122]
Pacritinib	JAK2, FLT3, IRAK1, CSF1R, and ACVR1	MF with platelet count <50 K/μL [122]
Momelotinib	JAK1/2, and ACVR1	Approved for intermediate- and high-risk PMF or SMF with anemia [123]
Drug Combinations (+/−JAKi) with promising clinical trial results		
Panobinostat	HDAC	Synergistically induce apoptosis [142,143]
IMG7289 (Bomedemstat)	LSD1	Synergize with ruxolitinib, selectively inhibit the *ASXL1*-mutant clone [144,147]
Pelabresib	BET	Lower inflammatory cytokines, act on megakaryocyte differentiation and proliferation [149,150,151,160]
C220, PRT543	PRMT5	Lower the mutation burden, reducing blood cell counts, and spleen size [153,154]
Imetelstat (monotherapy)	telomerase	Better response in patients refractory to JAKi, and harboring additional *SF3B1* and *U2AF1* mutations [155,156,157]
Navitoclax ABT-737	BCL2	Synergize with a JAKi, overcome acquired ruxolitinib resistance [158,159]
Enasidenib	IDH2	Only for patients with *IDH2* mutation [161]
Pegifna (+JAKi or HMA)	Inhibitor of hematopoietic cell proliferation; targeting progenitors carrying the *JAK2* V617F mutation	Patients with *SRSF2* or *ASXL1* mutations did not respond well to IFN-α [169,170,171,172,173].
Pomalidomide (monotherapy)	Immune modulator	Response not affected by additional (HMR) mutations.

HMA: hypomethylating agent; HMR: high molecular risk; JAKi: JAK inhibitor; Pegifna: pegylated IFN-α; PMF: primary myelofibrosis; SMF: secondary myelofibrosis; US FDA: United States Food and Drug Administration.

## 4. Conclusions and Future Directions

The traditional phenotype-based classification of MPNs lacks full recognition of their underlying pathobiology. The rapid accumulation of mutation profiling data in clinical patients has transformed our understanding of MF. Recent advances in single-cell sequencing technology have enabled a more accurate exploration of the clonal architecture and dynamics of neoplastic cell evolution, facilitating a better understanding of disease progression mechanisms. Additionally, studies focusing on epigenetic changes, transcriptional modifications, and the role of the microenvironment in disease pathogenesis and progression have begun to provide information on previously poorly understood aspects of MF. Considering the challenges in distinguishing ET from prefibrotic PMF [175,176] and the reported clinical cases in which typical prefibrotic megakaryocytes were not associated with disease progression for extended periods [176], the integration of genetic and mutation profiles for the subclassification of MPNs appears to be both beneficial and biologically reasonable [25,177]. However, many unanswered questions regarding the pathobiology and clinical management of MF persist. It is still uncertain whether PMF and SMF represent the same disease process with similar molecular signatures or a culmination of distinct disease processes while presenting with a shared phenotype of MF at the late stage.

Looking ahead, utilizing larger panels, including WES or WGS supplemented with WTS, to identify more clinically significant genetic alterations could help identify additional drivers, particularly of TN MPN. Personalized risk prediction to guide treatment, driver mutation-specific targeted therapies, and modifications of treatment protocols based on mutation profiles are desirable. The ultimate goal is to achieve tailored risk prediction for individual patients and to devise disease biology-based treatment protocols for curative therapies. The prospect of specific therapies that induce complete molecular genetic remission, analogous to tyrosine kinase inhibitor therapy for *BCR::ABL1*-postiive CML, remains a compelling approach in the pursuit of the best clinical outcomes for MF.

## Figures and Tables

**Table 2:** Prognostic systems for MPN and MF that include molecular genetic factors for scoring [113].

Prognostic Model	Karyotype or Mutations Included in the Score Calculation (Score points)	Risk Groups (OS)
MIPSS70 + v2.0 [107]	Non-*CALR* type 1 (2) HMR = 1 (2), HMR ≥ 2 (3), HR karyotype (3), VHR karyotype (4)	(Median OS) Very low (not reached) Low (16.4 year) Intermediate (7.7 year) High (4.1 year) Very high (1.8)
MPN Personalized Risk Calculator [25] (not for MF only)	Mutations in 33 genes Cytogenetic abnormalities	Individualized risk calculator
Myelofibrosis Secondary to PV and ET- Prognostic Model (MYSEC-PM) [112]	*CALR*-unmutated genotype	(Median OS) Low risk (not reached) Intermediate-1 (9.3 year) Intermediate-2 (4.4 year) high risk (2 year)
MTSS [114]	Non *CALR*/*MPL* (2) *ASXL1* (1)	(5-year OS) Low (83%) Intermediate (64%) High (37%) Very high (22%)

HMR: High molecular risk mutations, including mutations in *ASXL1*, *SRSF2*, *EZH2*, *IDH1/2*, or *U2AF1* Q157; HR: high risk; MF: myelofibrosis; MIPSS70 + v2.0: Mutation-Enhanced International Prognostic Scoring System plus version 2.0; MPN: myeloproliferative neoplasm, *BCR::ABL1* negative; MTSS: Myelofibrosis Transplant Scoring System; OS, overall survival; VHR: Very high risk. See text for details of HR and VHR chromosome abnormalities. MIPSS70 + v2.0 Online calculator: https://www.mipss70score.it (accessed on 30 December 2023). MPN Personalized Risk Calculator Online calculator: https://blood.predict.nhs.uk/ (accessed on 30 December 2023). https://www.sanger.ac.uk/science/tools/progmod/progmod/ (accessed on 30 December 2023). MYSEC-PM Risk Calculator: http://www.mysec-pm.eu/ (accessed on 30 December 2023).

## Abbreviations

*ABL1*	Abelson murine leukemia viral oncogene homolog 1
ACVR1	Activin A receptor, type I
*ASXL1*	Additional Sex Combs Like 1
BAPN	β-aminopropionitrile
*BCR*	Breakpoint cluster region
BET	Bromodomain and Extra-Terminal
*CBL*	Casitas B-lineage lymphoma proto-oncogene
*CDKN2A*	Cyclin-dependent kinase inhibitor 2A
*CRISPR*/Cas9	Clustered regularly interspaced palindromic repeats/CRISPR-associated protein 9
CSF1R	Colony stimulating factor 1 receptor
*CUX1*	Cut like homeobox 1
*DNMT3A*	DNA (cytosine-5)-methyltransferase 3A
*ETV6*	ETS (E-twenty-six) variant transcription factor 6
*EZH2*	Enhancer of zeste homolog 2
FLT3	fms-like tyrosine kinase 3
G6B	Megakaryocyte and platelet inhibitory receptor G6b
HDAC	Histone deacetylase
*IDH*	Isocitrate dehydrogenases
IL	Interleukin
IRAK1	Interleukin-1 receptor-associated kinase 1
*JAK2*	Janus Kinase 2
*KRAS*	Kirsten rat sarcoma virus (oncogene)
*LOX*	Lysyl Oxidase
LSD1	Lysine-specific demethylase-1
*MPL*	Myeloproliferative leukemia proto-oncogene
*NFE2*	Nuclear factor erythroid 2
NF-κB	Nuclear factor kappa-light-chain-enhancer of activated B cells
*NOTCH1*	Neurogenic locus notch homolog protein 1
*NRAS*	Neuroblastoma RAS viral oncogene homolog
PRMT5	Protein arginine methyltransferase 5
*RUNX1*	Runt-related transcription factor 1
SAP	Serum amyloid P
*SF3B1*	Splicing Factor 3b, Subunit 1
*SH2B3*	Src-homology 2B adapter protein 3
*SRSF2*	Serine-arginine splicing factors 2
STAT	Signal transducer and activator
*TET2*	Ten-Eleven Translocation 2
*TP53*	Tumor protein p53
*U2AF1*	U2 small nuclear RNA auxiliary factor 1
*ZRSR2*	Zinc Finger CCCH-Type, RNA Binding Motif and Serine/Arginine Rich 2
